# Columbia Veterinary College, Opening of the Sixth Session

**Published:** 1884-01

**Authors:** 


Columbia Veterinary College—Opening of the Sixth Session.—Dr. Hubbard W. Mitchell delivered an address entitled "The Importance of the Collateral Branches of Science to Comparative Medicine," in the course of which he made the following remarks:

"You have chosen the department of comparative medicine for your life vocation, and as you advance in it you will find a great and important truth gradually dawning upon you, telling you that broad as our department of medicine seems to us, it is but one of many in that vast science of biology which teaches us of every form of life, from the tiny animalcule, invisible to the unaided eye, through the whole range of the animal kingdom, instinct with exhuberant and glowing activity up to the higher and more intelligent species of which we ourselves form a part." * * * * * * *

"The veterinarian of to-day is immensely in advance of his confreres of even a few years ago. Then he was rude, often illiterate, crude in his methods, and with a scanty knowledge of the most common ailments of a single species of animal. He was looked upon with distrust and regarded with contempt. Now he is scientifically educated; he is a comparative practictioner. He has studied those sciences which are so necessary to a complete modern education and he is fitted to take a higher place in his profession.

The collateral branches of science bear very closely upon each other, and the student must know them if he would be up to his time. His study of meteria-medica demands a knowledge of botany, while that of surgery demands a knowledge of pathology, histology, and morbid anatomy. He cannot fully and clearly comprehend the wondrous study of physiology which treats of the functions of life unless he also understands something of biology." * * * * *

"As doctors of comparative medicine you have a wide field before you. There are in this country about *one hundred and twenty millions* of domestic animals, having a value of about two billion dollars. Besides this there are the numerous collections of wild animals, and to you, gentlemen, is confided the care and well-being of this mighty swarm of animal life. You will be called upon to treat disease in many forms among this stupendous living herd, and when you remember that you stand as a barrier between this great mass of animal life and *fifty million human beings* to prevent disease from passing from one to the other, you will then appreciate the importance of your mission."

